# UBE2C Is a Potential Biomarker of Intestinal-Type Gastric Cancer With Chromosomal Instability

**DOI:** 10.3389/fphar.2018.00847

**Published:** 2018-08-02

**Authors:** Jun Zhang, Xinyu Liu, Guanzhen Yu, Lei Liu, Jiejun Wang, Xiaoyu Chen, Yuhai Bian, Yuan Ji, Xiaoyan Zhou, Yinan Chen, Jun Ji, Zhen Xiang, Lei Guo, Jingyuan Fang, Yihong Sun, Hui Cao, Zhenggang Zhu, Yingyan Yu

**Affiliations:** ^1^Department of Surgery, Ruijin Hospital, Shanghai Key Laboratory for Gastric Neoplasms, Shanghai Institute of Digestive Surgery, Shanghai Jiao Tong University School of Medicine, Shanghai, China; ^2^Changzheng Hospital, Affiliated to Second Military Medical University, Shanghai, China; ^3^Department of Oncology, Longhua Hospital of Shanghai University of Traditional Chinese Medicine, Shanghai, China; ^4^Renji Hospital, Affiliated to Shanghai Jiao Tong University School of Medicine, Shanghai, China; ^5^Zhongshan Hospital, Affiliated to Fudan University, School of Medicine, Shanghai, China; ^6^Cancer Hospital, Affiliated to Fudan University School of Medicine, Shanghai, China

**Keywords:** gastric cancer, Lauren classification, data mining, biomarkers, UBE2C

## Abstract

This study explored potential biomarkers associated with Lauren classification of gastric cancer. We screened microarray datasets on gastric cancer with information of Lauren classification in gene expression omnibus (GEO) database, and compared differentially expressing genes between intestinal-type or diffuse-type gastric cancer. Four sets of microarray data (GSE2669, GSE2680, GDS3438, and GDS4007) were enrolled into analysis. By differential gene analysis, UBE2C, CDH1, CENPF, ERO1L, SCD, SOX9, CKS1B, SPP1, MMP11, and ANLN were identified as the top genes related to intestinal-type gastric cancer, and MGP, FXYD1, FAT4, SIPA1L2, MUC5AC, MMP15, RAB23, FBLN1, ANXA10, and ADH1B were genes related to diffuse-type gastric cancer. We comprehensively validated the biological functions of the intestinal-type gastric cancer related gene UBE2C and evaluated its clinical significance on 1,868 cases of gastric cancer tissues from multiple medical centers of Shanghai, China. The gain of copy number on 20q was found in 4 out of 5 intestinal-type cancer cell lines, and no similar copy number variation (CNV) was found in any diffuse-type cancer cell line. Interfering UBE2C expression inhibited cell proliferation, migration and invasion *in vitro*, and tumorigenesis *in vivo*. Knockdown of UBE2C resulted in G2/M blockage in intestinal-type gastric cancer cells. Overexpression of UBE2C activated ERK signal pathway and promoted cancer cell proliferation. U0126, an inhibitor of ERK signaling pathway reversed the oncogenic phenotypes caused by UBE2C. Moreover, overexpression of UBE2C was identified in human intestinal-type gastric cancer. Overexpression of UBE2C protein predicted poor clinical outcome. Taken together, we characterized a group of Lauren classification-associated biomarkers, and clarified biological functions of UBE2C, an intestinal-type gastric cancer associated gene. Overexpression of UBE2C resulted in chromosomal instability that disturbed cell cycle and led to poor prognosis of intestinal-type gastric cancer.

## Introduction

Gastric cancer is one of the most common digestive malignancies, which threats public health worldwide, especially in Asia (Torre et al., 2015). Lauren classification is a standard histopathological classification which divides gastric cancer into two subtypes: intestinal-type and diffuse-type (Lauren, 1965). This histological classification was established based on histological observation by a Nordic pathologist half a century ago when there was no evidence at molecular level. Multiple reports including our previous study had demonstrated that there are different biological behaviors between intestinal-type and diffuse-type gastric cancers (Liu et al., 2013; Qiu et al., 2013; Chen et al., 2016). However, there is no systematic study on biomarkers that associated with Lauren classification in gastric cancer.

With the application of high-throughput gene analysis, many gene expression profiles of gastric cancer have been accumulated and deposited in open accessible database. DNA microarray or gene chip is the first one of the high-throughput technologies applied on molecular classification for cancers (Tusher et al., 2001). HER2/Neu positive breast cancer was successfully identified according to microarray analysis (Slamon and Pegram, 2001; van 't Veer et al., 2002). Utilization of high-throughput technologies also promoted changing gastric cancer research from histologic level to molecular level (Ji et al., 2002; Kim et al., 2003). Currently, a number of datasets come from separated institutes and there is no comparable analysis.

In this study, microarray datasets of gastric cancer deposited in NCBI database were extracted, and microarray data with clear Lauren classification information were enrolled into data mining. We found a set of Lauren classification-associated differential genes. UBE2C is one of the genes that highly expressed in intestinal-type gastric cancer. We validated the expression of UBE2C on a separated cohort of gastric cancer from Shanghai, China. To clarify the underlying molecular mechanisms of UBE2C, we explored the variation of UBE2C gene copy number in gastric cancer cells originated from intestinal-type or diffuse-type gastric cancers. We further analyzed biological function and related signaling pathway of UBE2C gene both *in vitro* and *in vivo*. This study established a link between up-regulation of UBE2C with chromosome instability and disturbed ERK signaling pathway in intestinal-type gastric cancer.

## Materials and methods

### DNA microarray data analysis

We searched gene expression omnibus (GEO) database of NCBI (http://www.ncbi.nlm.nih.gov/geo/) using keywords of “Gastric Cancer,” “Gastric Carcinoma” or “Stomach cancer,” and obtained four sets of microarray datasets (GEO serial numbers: GSE2669, GSE2680, GSE3438, and GSE4007) that included detail information of Lauren classification. Significance Analysis of Microarray (SAM) was used for differential genes selection of intestinal-type gastric cancer and diffuse-type gastric cancer through an open-accecerable tool, MultiExperiment Viewer (MEV) which supported by Stanford University (Tusher et al., 2001; Saeed et al., 2003). By adjusting permutation, false disvovery rate (FDR) and delta value, we obtained sets of differential genes between intestinal-type gastric cancer and nomal mucoasa, diffuse-type gastric cancer and normal mucosa, as well as differential genes between intestinal-type and diffuse-type gastric cancers.

### Tissue microarray construction and immunohistochemistry

This study was approved by the institutional review board of hospital, and a written informed consent was obtained from the participants of this study. A total of 1,868 cases of gastric cancer were included in this study. All cancer tissues came from gastrectomy in the following 4 medical centers of Ruijin hospital, Renji hospital, Zhongshan hospital, and Changzheng hospital, Shanghai, China from 2002 to 2015. Including criteria were: patients with histological slides and paraffin blocks. Excluding criteria were: patients who have stump gastric cancer, neoadjuvant chemotherapy, incomplete information, multiple lesions, and combined with other malignancies. Finally, 109 cases were excluded and only 1,759 cases were included in the final analysis. Among them, 1,224 cases (69.6%) were male, and 535 (30.4%) cases were female. The median age was 61-year old (22–88 year-old). All samples were fixed in neutral buffered formalin and embedded in paraffin. Patients were followed up every 6 months for the first 3 years and annually after 3 years. The last followed up was December 30, 2015, with the longest follow-up time of 142 months. The average follow-up time was 48.73 months. Tumor stage was determined based on the 7th edition of TNM staging system by AJCC/UICC organizations.

Tissue microarray blocks were constructed from these 1,759 cases using a tissue microarrayer (Beecher Instruments, Silver Spring, MD, USA). Two tissue cores (2 mm diameter) from each cancer or normal mucosa block were taken and transferred to a recipient paraffin block. Subsequently, 4-mm-thick sections were cut from each tissue microarray block, de-waxed, and dehydrated.

A standard peroxidase-conjugated streptavidin-biotin method was used for immunohistochemical staining with UBE2C (mouse-anti human UBE2C monoclonal antibody, 1:50, SC-100611, Santa Cruz, SC-100611, USA). The immunohistochemical staining results were evaluated by two independent pathologists and scored according to the percentage of positive cells and staining intensity. The graded percentage of positive cells 0 (<10%), 1 (10–30%), 2 (31–50%), and 3 (51–100%) was recorded. The staining intensity was graded as 0 (negative), 1 (weak), 2 (moderate), and 3 (strong). A cumulative score ranging from 0 to 9 was obtained by multiplying the staining intensity and percentage. The total score graded as weakly positive (scores <3) and moderately or strongly positive (scores ≥4).

### Analysis of chromosomal copy number in gastric cancer cells

The global copy number variation (CNV) of chromosomes was analyzed in 6 different cancer cell lines: SGC-7901, BGC-823, MKN-45, MKN-28, AGS, and HTB-103 by Affymetrix® Genome-Wide Human SNP Array 6.0 chip. The peripheral blood samples from 2 healthy individuals were used as controls. The information of gain or loss of copy number of each sample was analyzed by Genotyping Console software, which was calculated based on comparison of Hapmap project.

### Cell culture

Cancer cells were seeded in 6-well plates (3.0 × 10^5^ cells) and cultured in RPMI-1640 medium with 10% fetal bovine serum (Gibco, Invitrogen, USA) in 5% CO_2_ cell culture incubator at 37°C. Transfection of siRNAs or eukaryotic expression vectors was carried out using Lipofectamine 2000 (Invitrogen, Carlsbad, CA) according to protocol provided with the product. The cells were harvested 3–5 days following transfection.

### Western blot

The protein concentration was determined using a BCA Kit (Pierce Biotechnology, Rockford, IL, USA). Each protein extract (100 μg) was electrophoresed on a 12.5% SDS-polyacrylamide gel, transferred to PVDF membranes in a buffer containing 25 mMTris-HCl (pH 8.3), 192 mM glycine and 20% (v/v) methanol, and blocked in 5% (w/v) skimmed milk in Tris buffered saline-Tween 20 (0.1% by volume, TBST) for 2 h at room temperature, and probed with specific primary antibodies overnight at 4°C. Blots were reacted with secondary antibodies coupled to horseradish peroxidase in TBST. The primary antibodies were mouse anti-UBE2C (1:1,000; Cat. AM1831a, ABGENT, San Diego, CA, USA), anti-ERK (Cat. 4695S, Cell Signaling Biotechnology), p-ERK (1:1,000; Cat. 9284, Cell Signaling Biotechnology) and mouse anti-glyceraldehyde-3-phosphate dehydrogenase (1:5,000, Sigma) antibody. The horseradish peroxidase-conjugated secondary antibodies were goat anti-rabbit IgG (1:2,000; Cat. 7,074, Cell Signaling Biotechnology) or horse anti-mouse IgG (1:2,000; Cat. 7,074, Cell Signaling Biotechnology). Signals were detected by a SuperSignal West Pico Chemiluminescent Substrate kit (Pierce, Rockford, IL, USA) according to the manufacturer' s instructions.

### Cell proliferation assay

CCK8 assay was performed to assess the effect of UBE2C on cell proliferation. Cells were transfected with siRNAs or UBE2C overexpressing vector for approximately 12 h. Following transfection, cells were transferred to 96-well microplates and seeded at a density of approximately 1.0 × 10^3^ cells per well. Then each batch of cells were stained with 10 μl of CCK8 regent (Dojindo, Kumamoto, Japan) at 37°C for 2 h every 24 h for 4 days. The coloring reaction was quantified by an automatic plate reader (Tecan, Swiss) at 450 nm. All experiments were performed in triplicate.

For clonogenicity assay, cells were transfected with siRNAs or UBE2C overexpressing vector for approximately 12 h. Following transfection, cells were transferred into 6-well plates and seeded at a density of approximately 1.0 × 10^3^ cells per well. Culture medium was changed every 3 days. Clonogenicity was analyzed 12 days following transfection by staining cells with 0.05% crystal violet solution for 1 h. Visible colonies were manually counted. A group of cells >50 was counted as a colony. The data were reported as means ± SD by counting 10 fields randomly.

### Cell migration and invasion

For migration and invasion assays, cell culture was performed in 24-well Transwell chambers (Corning, USA). For the invasion assay, the insert membranes were coated with diluted Matrigel (BD Biosciences, USA). Cells (1 × 10^5^) were added to the upper chamber and cultured for 48 h. For the migration assay, cells were cultured under the same conditions except that the insert membranes were not coated with Matrigel. Finally, the insert membranes were cut and stained with 0.1% crystal violet for 2 min. The penetrating cells were counted under an inverted microscope and photographed in five random fields and the average number of cells per field was counted. These experiments were performed in triplicate.

### Construction of shRNA and shUBE2C plasmids

The full-length cDNA of UBE2C (NM_000690.3) was purchased (GENECHEM, Shanghai, China). The full-length cDNA of human UBE2C was cloned and inserted into pBABE-puro vector (Clontech Laboratories, Mountain View, CA, USA). Two pairs of complementary short hairpin RNA (shRNA) expression vectors targeting UBE2C (UBE2C-shRNA1 and UBE2C-shRNA2) and a pair of scrambled negative control shRNA oligonucleotides were constructed using RNAi-Ready pSIREN-RetroQ vector (Clontech Laboratories). The retroviral packaging process was performed in a plate of 293T cells. After 12 h, 15 mL viral collection medium was added into the transfected cells. Infection of BGC-823 or SGC-7901 cancer cells was performed in the presence of 5 μg/mL of polybrene (Sigma, MO, USA) in each well of a 6-well plate. Stable retroviral transduction was achieved by infection for 48 h and followed by puromycin (1 μg/mL) selection.

### Assay of ERK signaling pathway

The ERK1/2 inhibitor U0126 was purchased from Selleckchem (Cat. 9903S). The final concentration of U0126 used in culture medium was 10 μmol/L. Cells were harvested for protein extraction and Western blotting analysis.

### Tumorigenesis of UBE2C in nude mice

Cancer cells (1 × 10^6^/100 μl) transfected with UBE2C or sh-UBE2C were collected and inoculated subcutaneously into 4-week-old BALB/c nude mice (Institute of Zoology, Chinese Academy of Sciences, Shanghai). Each experimental and control groups consisted of four mice. Tumor nodules were measured every 4 days with a caliper. Mice were sacrificed after 1 month. Tumor growth curves and inhibiting rates were calculated. After tumor excision, the tissues were fixed in 10% buffered formalin. All formalin-fixed and paraffin-embedded samples were carefully examined after staining with hematoxylin-eosin (HE) and photographed. The animal experiment was approved by the Institutional Animal Care and Use Committee of Shanghai Jiao Tong University School of Medicine.

### Statistical analysis

Statistical analysis was performed using SPSS 17.0 statistical software (SPSS Inc., Chicago, IL, USA.). Associations between expression of UBE2C and clinicopathological variables were analyzed using the Chi square test. The continuous variables were expressed as mean values ± standard deviation. The differences between groups were analyzed using the Student's *t-*test when there were only two groups, or assessed by one-way ANOVA when there were more than two groups. Two-way ANOVA was used for the analysis of multiple comparisons. Cox regression method was used to analyze multivariate survival. Kaplan-Meier method and Log-Rank test were used to analyze non-parametric survival. A *P*-value of <0.05 was considered statistically significant.

## Results

### Data mining revealed genes related to lauren classification

By searching GEO database, datasets GSE2669, GSE2680, GSE3438, and GSE4007 were selected because they contain large sample size with detail clinical information including Lauren classification of gastric cancer (Table 1). We extracted differential expression genes between intestinal-type vs. control or diffuse-type vs. control by SAM method. The permutation value was set at 1,000 and FDR <5% on 1.5-fold-change. A total of 263 feature genes were chacaterized. Among them, 40 genes were highly expressed in intestinal-type gastric cancer, and 223 genes were highly expressed in diffuse-type gastric cancer. We listed the top 10 genes with >1.5-fold-change in two types of gastric cancers (Table 2).

**Table 1:** The details of four microarray datasets from GEO database.

**GEO ID**	**Controls**	**Case No**.	**Samples**	**Submitter**
GSE2669	Mixture of normal samples	124	64 cancer 26 gastritis 22 metaplasia 10 normal 2 other	Boussioutas A, National Cancer Centre of Singapore
GSE2680	Mixture of normal samples	90	68 intestinal- 13 diffuse- 9 mixed-	Aggarwa A, National Cancer Centre of Singapore
GSE3438	Exchange of marked staining reagents	100	50 cancer 50 normal	Kim S, Korea Research Institute of Bioscience and Biotechnology
GSE4007	Mixture of normal samples	126	90 cancer 22 normal 14 lymph node	Chen X, Stanford University

**Table 2:** The top 10 related genes in intestinal- or diffuse-type gastric cancer.

**Gene symbol**	**Gene names**	**Chromosome loci**
**INTESTINAL-TYPE**		
**UBE2C**	Ubiquitin-conjugating enzyme E2C	20q13.12
CDH1	Cadherin 1, type 1, E-cadherin (epithelial)	16q22.1
ERO1L	ERO1-like (*S. cerevisiae*)	14q22.1
SCD	Stearoyl-CoA desaturase (delta-9-desaturase)	10q24.31
SOX9	SRY (sex determining region Y)-box 9	17q24.3-q25.1
CKS1B	CDC28 protein kinase regulatory subunit 1Apseudogene	8q21.13
SPP1	Osteopontin	4q21-q25
MMP11	Matrix metallopeptidase 11 (stromelysin 3)	22q11.2|22q11.23
CENPF	Centromere protein F, 350/400 ka (mitosin)	1q32-q41
ANLN	Anillin, actin binding protein	7p15-p14
**DIFFUSE-TYPE**		
FAT4	FAT tumor suppressor homolog 4 (Drosophila)	4q28.1
SIPA1L2	Signal-induced proliferation-associated 1 like 2	1q42.2
RAB23	RAB23, member RAS oncogene family	6p11
FBLN1	Fibulin 1	22q13.31
ADH1B	Alcohol dehydrogenase 1B (class I), beta polypeptide	4q21-q23
ANXA10	Annexin A10	4q33
MGP	Matrix Gla protein	12p13.1-p12.3
MUC5AC	Mucin 5AC, oligomeric mucus/gel-forming	11p15.5
FXYD1	FXYD domain containing ion transport regulator 1	19q13.1
MMP15	Matrix metallopeptidase 15 (membrane-inserted)	16q13-q21

### Up-regulation of UBE2C related to amplification of chromosomal copy number

**Figure 1:**
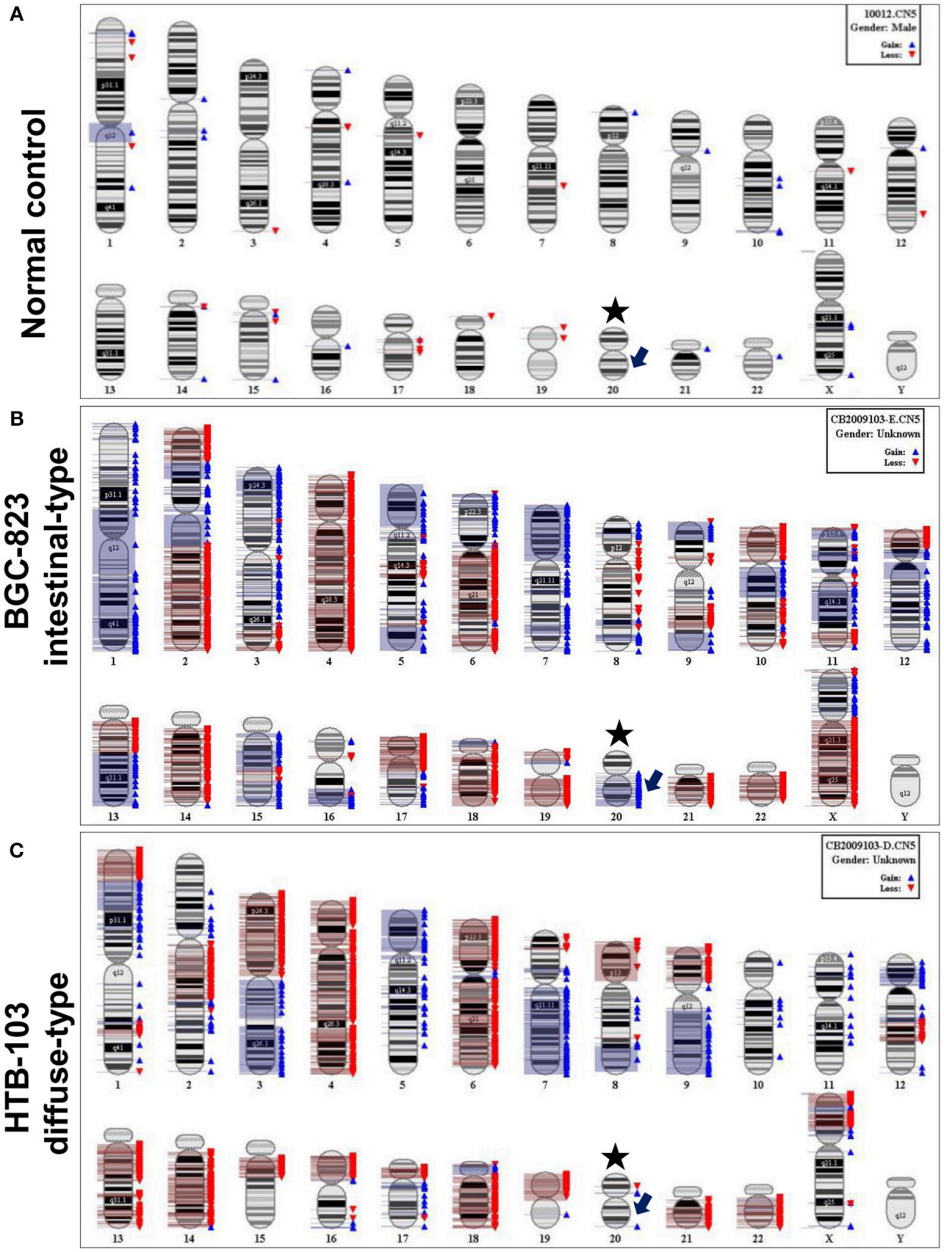
Representative chromosomal CNV features by SNP 6.0 microarray analysis. **(A)** Normal control from healthy peripheral blood. Asterisk indicates chromosome 20, and arrow indicates gene locus of UBE2C. **(B)** CNV feature of intestinal-type gastric cancer cell line BGC-823. The blue bar on the right of chromosome represents copy number amplification, and red bar means copy number loss. The arrow indicates copy number amplification in gene locus of UBE2C. **(C)** CNV feature of diffuse-type gastric cancer cell line HTB-103. There is no copy number amplification in gene locus of UBE2C.

Gastric cancer cell lines SGC-7901, BGC-823, MKN-45, MKN-28, HTB-103, and AGS were used to analyze chromosomal CNV. Four out of 5 intestinal-type gastric cancer cell lines BGC-823, MKN-45, MKN-28, and AGS revealed amplification of chromosome 20q which contains the gene locus of UBE2C. No 20q amplification was observed in diffuse-type gastric cancer cell line HTB-103 (Figure 1).

### Interfering or enforcing UBE2C expression affected cell growth and invasion

**Figure 2:**
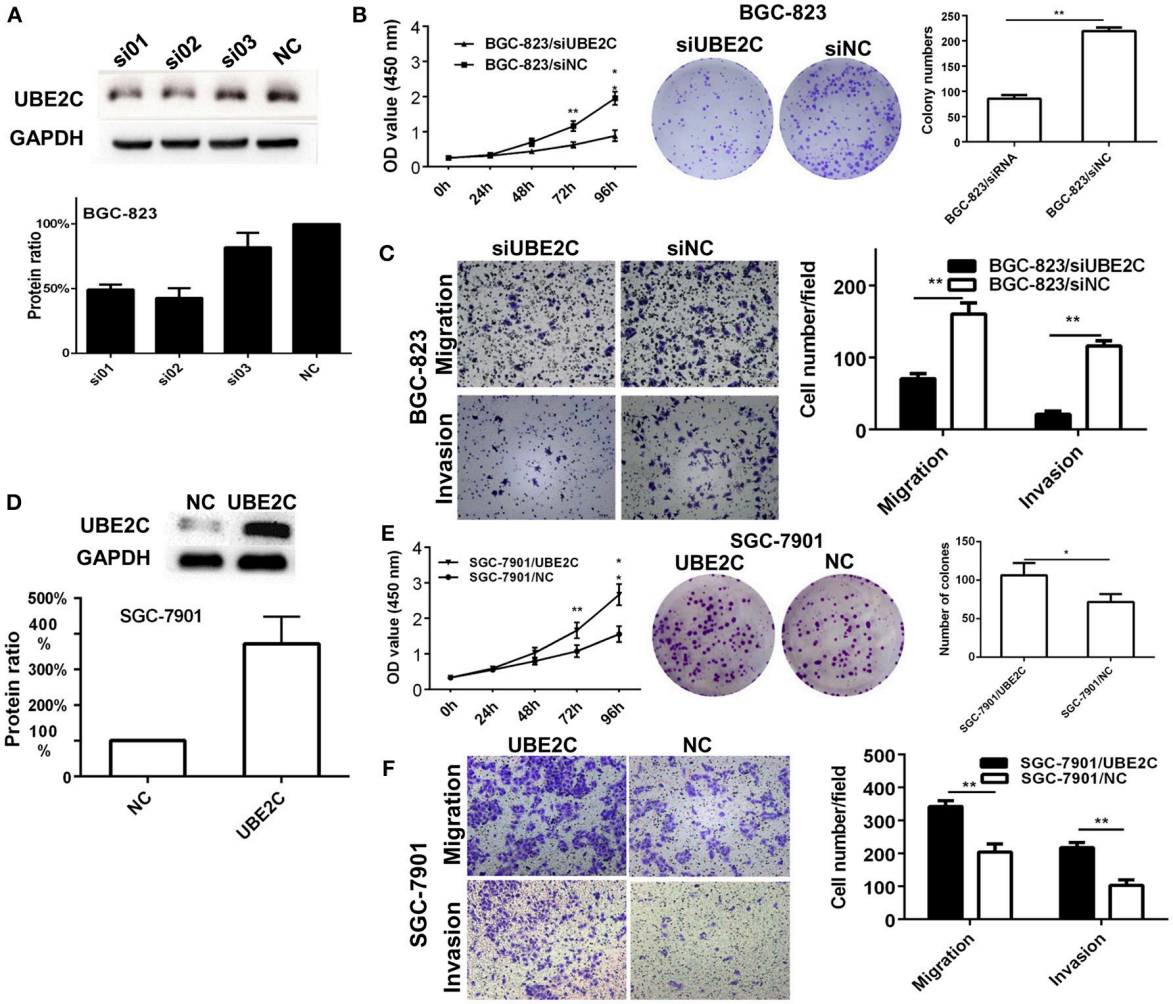
Interfering or enforcing UBE2C expression affected cancer cell proliferation and invasion. **(A)** Protein level of UBE2C detected after siRNA transfection in BGC-823 cells. Among three siRNA sequences, si02 showed the best knockdown efficacy. **(B)** Knockdown of UBE2C decreased cell growth and colony formation in BGC-823 cells. **(C)** Knockdown of UBE2C decreased migration and invasion of BGC-823 cells. **(D)** Protein level of UBE2C detected after enforcing UBE2C expression in SGC-7901 cells. **(E)** Overexpression of UBE2C increased cell growth and colony formation in SGC-7901 cells. **(F)** Overexpression of UBE2C increased migration (upper) and invasion (lower) of SGC-7901cells. Experiments were performed in triplicates. *p < 0.05; **p < 0.01.

Two intestinal-type gastric cancer cell lines, BGC-823 which has high expression of UBE2C and SGC-7901 which has low expression of UBE2C, were used to investigate the function of UBE2C on cell growth and invasion. The expression of UBE2C was monitored by qRT-PCR and Western blot. Cell proliferation was determined by CCK-8 assay and colony formation. The migration and invasion of cancer cells were determined by transwell assay. The UBE2C knockdown using si01 and si02 sequences achieved over 50% down-regulation of UBE2C. UBE2C-si02 was used for subsequent experiments because it down-regulated UBE2C expression by up to 57.24 ± 7.54% (Figure 2A). The siRNA was transfected into BGC-823 cells, and the light absorbance of 0, 24, 48, 72, and 96 h at 450 nm wavelength was determined. Compared to the siNC, the light absorbance of 72 and 96 h of siUBE2C was 0.62 ± 0.08 vs. 1.15 ± 0.14 (*P* = 0.001) and 0.88 ± 0.16 vs. 1.96 ± 0.19 (*P* = 0.001; Figure 2B). As for colony formation assay, colony numbers were evaluated at the 21st day after siUBE2C transfection. The colony numbers were significantly reduced in siUBE2C group, compared to siNC group (85 ± 12 vs. 219 ± 12, *P* = 0.001; Figure 2B). These results indicated that down-regulation of UBE2C inhibited cancer cell growth *in vitro*.

To examin the effect of UBE2C on cell migration and invasion abilities, transwell chambers were used. Migrated cells were counted after 48 h transfection of siUBE2C. The number of migrated cells decreased in siUBE2C group compared with the siNC group (71 ± 7 vs. 160 ± 16, *P* = 0.004; Figure 2C, upper panel). Similarly, the number of invasive cells decreased in siUBE2C group compared with the siNC group (21 ± 4 vs. 116 ± 7, *P* = 0.001, Figure 2C).

We reversely verified the functions of UBE2C by enforcing UBE2C expression in gastric cancer cells. The transfection efficacy of UBE2C eukaryotic expression vector was confirmed at 48 h after UBE2C transfection in SGC-7901 cells by Western blot. Compared to control, transfection of UBE2C increased protien level of UBE2C by 3.72 ± 0.75-folds (Figure 2D, *P* = 0.025). CCK-8 assay was used to examine cell proliferation. The 450 nm absorbance at 72 and 96 h for UBE2C and control group was 1.66 ±0.22 vs. 1.07±0.17 and 2.66±0.29 vs. 1.56±0.22 (Figure 2E), respectively. The ability of colony formation was evaluated in the UBE2C transfected and control cells after 21 days of UBE2C transfection. The colony numbers were significantly increased in UBE2C group, compared to the control group (106 ± 16 vs. 71 ± 11, *P* = 0.043, Figure 2E). These results supported that up-regulation of UBE2C promoted cell growth of cancer cells *in vitro*.

In addition, we analyzed the ability of cell migration and invasion at 48 h after enforcing UBE2C expression. There were increased migraed cells and increased invasive cells in the UBE2C group compared with control group (342 ± 18 vs. 204 ± 25, *P* = 0.002; Figure 2F, upper panels) and (218 ± 16 vs. 103 ± 17, *P* = 0.001), (Figure 2F, lower panels). These results further confirmed that overexpression of UBE2C promoted invasive ability of gastric cancer cells *in vitro*.

### Down-regulation of UBE2C caused cell cycle arrest and inhibited ERK1/2 signaling pathway

**Figure 3:**
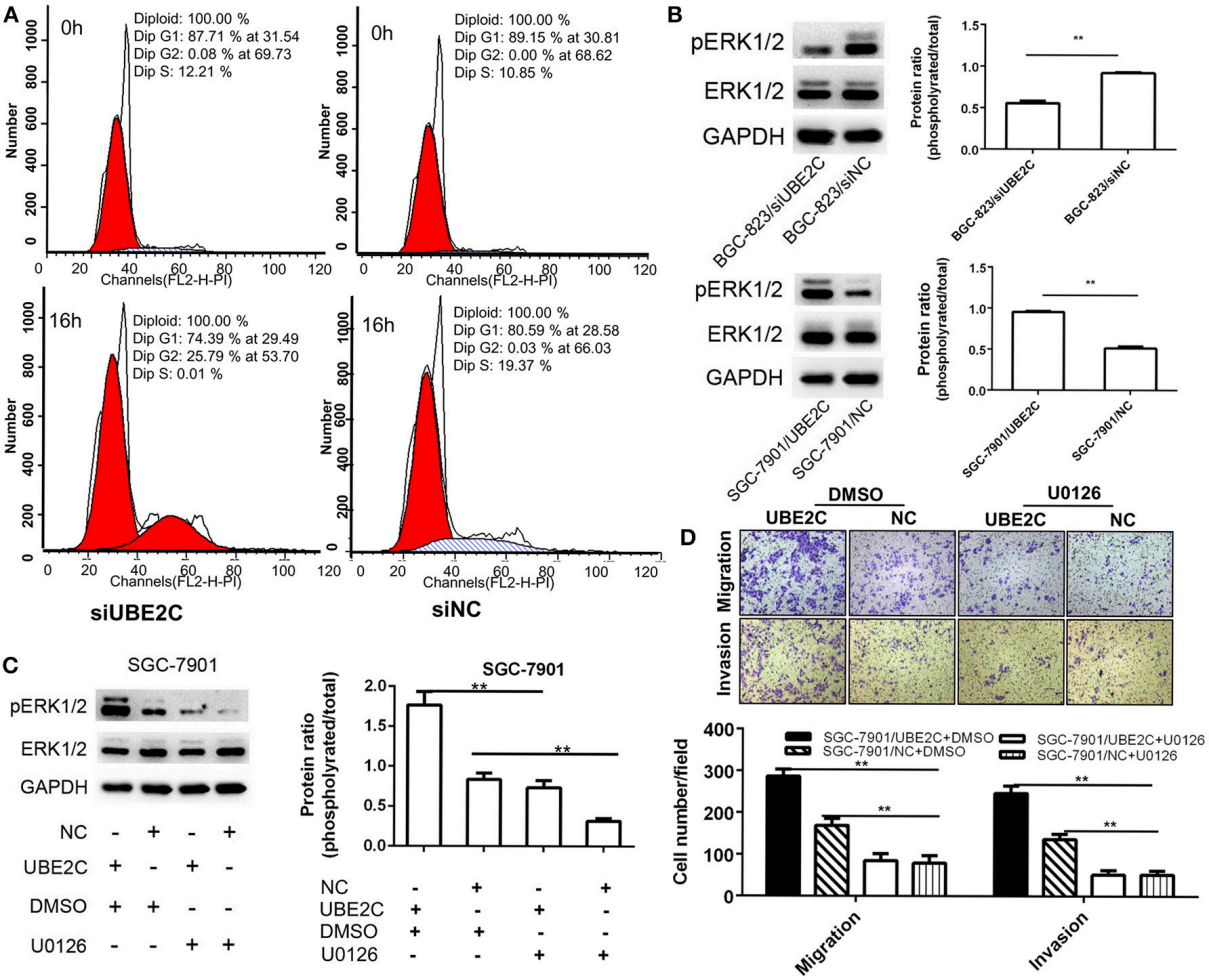
Cell cycle and ERK1/2 signaling pathway affected by interfering or enforcing UBE2C expression. **(A)** Cell cycle detected after release of thymidine blockage at 0 and 16 h. The percentage of cells in G_2_/M phase was significantly higher (25.79%) in siUBE2C group than that in siNC group (0.03%). **(B)** Knockdown of UBE2C decreased ERK1/2 phosphorylation and overexpression of UBE2C promoted ERK1/2phosphorylation. **(C)** U0126 reversed phosphorylated ERK1/2 level caused by UBE2C overexpression. **(D)** Migration (upper) and invasion (lower) inhibited by U0126 treatment in SGC-7901 cells. Experiments were performed in triplicates. **p < 0.01.

Cell cycle was analyzed by synchronize cells using dual blockage of thymidine for 16 h. Flow cytometry analysis found that the G2/M fraction of BGC-823/siUBE2C group was significantly higher than those in control group (25.79±0.72 vs. 0.03±0.02%, *P* = 0.001), while the G1 fraction (74.39±0.72 vs. 80.59±2.32% *P* = 0.034) and S fraction (0.01 ± 0.01 vs. 19.37 ± 2.32%, *P* = 0.005) were less than those in controls (Figure 3A). This result suggested that about one-fourth of the cells was blocked in G2/M phase by down-regulation of UBE2C in gastric cancer cells.

The phosphorylation of extracellular signal-regulated kinase1/2 (ERK1/2) was analyzed by Western blotting. The results revealed that UBE2C silencing down-regulated the phosphorylation of the ERK1/2 in BGC-823 cancer cells, while UBE2C overexpression up-regulated phosphorylation of the ERK1/2 in SGC-7901 cancer cells (Figure 3B). Additionally, the ERK1/2 inhibitor U0126 was used to confirm the signaling pathway involved in UBE2C-mediated cancer progression. ERK1/2 inhibitor U0126 (10 μmol/L) decreased the phosphorylation of the ERK1/2 caused by UBE2C overexpressing SGC7901 cells. The ratio of phosphorylated ERK1/2 to ERK1/2 was 0.55 ± 0.03 vs. 0.92 ± 0.01, *P* = 0.001 (Figure 3C). On the contrary, the ratio of phosphorylated ERK1/2 to ERK1/2 increased, compared with control by UBE2C overexpression in SGC-7901 cells (0.95 ± 0.02 vs. 0.51 ± 0.02, *P* = 0.001).

ERK1/2 inhibitor U0126 (10 μmol/L) was added in culture medium and CCK-8 assay was used to examine cell proliferation. We found that the 450 nm absorbance decreased at 72 h (0.69 ± 0.14 vs. 1.61 ± 0.19, *P* = 0.003) and 96 h (0.82 ± 0.16 vs. 2.6 ± 0.32, *P* = 0.003) in UBE2C-U0126 group compared to UBE2C-DMSO group, respectively. This result suggested that reduced ERK1/2 phosphorylation could reverse cell proliferation induced by UBE2C.

We analyzed the ability of cell migration and invasion at 48 h after SGC-7901 cancer cells were incubated with U0126. Compared with control group, the migrated cells in UBE2C-U0126 group were significantly less than those in UBE2C-DMSO group (286 ± 17.52 vs. 84.33 ± 17.24, *P* = 0.001; Figure 3D, upper panel), while the invasive cells was also less in UBE2C-U0126 group than that in UBE2C-DMSO group (136.67 ± 12.5 vs. 51.67 ± 9.61, *P* = 0.001, Figure 3D, lower panel). These results confirmed that reduced ERK1/2 phosphorylation could reverse cell invasive ability *in vitro*.

### Intervening UBE2C affected tumorigenesis *in vivo*

**Figure 4:**
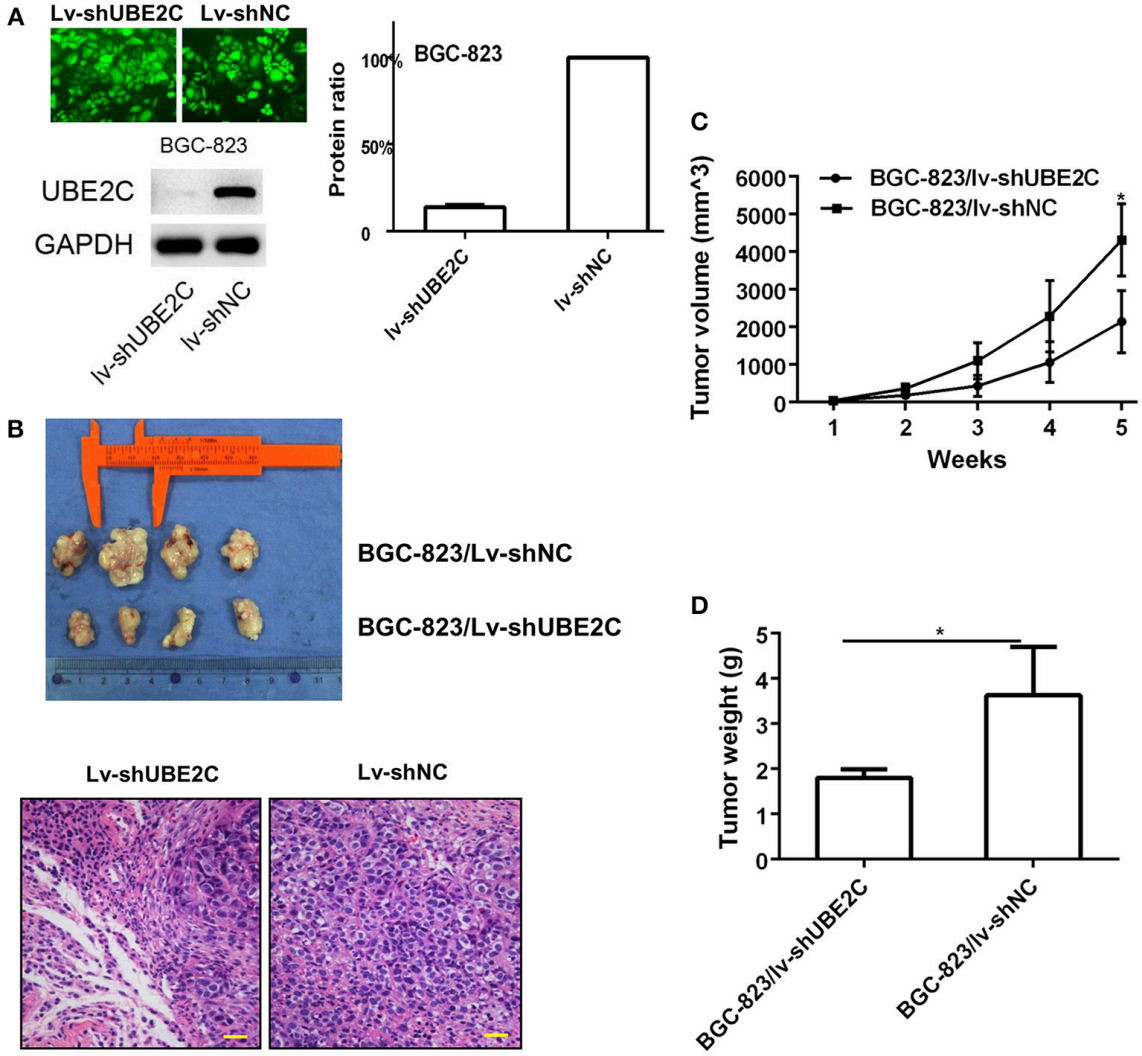
Effect of UBE2C in *in vivo* tumorigenesis. **(A)** Fluorescent images of stably transfected BGC-823 cells (200 × ). All cells were transfected. Knockdown of Lv-shUBE2C was documented by Western blot analysis. **(B)** Knockdown of UBE2C inhibited tumorigenesis in nude mice at day 35 after tumor cell inoculation. The tumor volumes were obviously larger in experimental group than that in control group (upper). Under microscope, tumor cells were reduced in experimental group, compared to control (down). Scale bar indicates 25 μm. **(C)** Tumor volume curves of xenografts in different groups of BGC-823 cancer cells. **(D)** Tumor weight of xenografts in different groups of BGC-823 cancer cells. *p < 0.05.

Short hairpin RNA (shUBE2C) expression vectors were constructed to silence UBE2C. Lentiviral vector was used to infect BGC-823 cells and the transfection efficacy reached 100% observed under a fluorescence microscope. The expression of UBE2C was monitored by Western blot analysis. Compared with control group, the UBE2C expression in Lv-shUBE2C cells declined 77.00 ± 8.81% (*P* = 0.004; Figure 4A). The effect of UBE2C in tumorigenesis was evaluated in BALB/c nude mice. BGC-823/Lv-shUBE2C cells (1.5 × 10^6^) and control cells were inoculated subcutaneously (*n* = 4). The tumor volumes were measured every week. Mice were sacrificed at the 35th day of tumor formation. It was found that the tumor volume was smaller in BGC-823/Lv-shUBE2C group compared with the BGC-823/Lv-shNC group. Under microscope, tumor cells were reduced in experimental group, compared to control (Figure 4B), while both tumor volume (2136.99 ± 827.98 vs. 4304.33 ± 958.75 mm^3^, *P* = 0.015) and tumor weight (1.8 ± 0.18 vs. 3.6 ± 1.07 g, *P* = 0.04) of BGC-823/Lv-shUBE2C group were decreased compared to those in BGC-823/Lv-shNC group (Figures 4C,D).

In addition, lentiviral vector of UBE2C was used to infect SGC-7901 cells which inoculated to nude mice (*n* = 4) subcutaneously. The tumor volumes were measured every week. Mice were sacrificed at the 35th day. Compared with the SGC-7901/Lv-NC group, the tumor volume was larger in SGC-7901/Lv-UBE2C group (2096.85 ± 944.66 vs. 3986.48 ± 1306.44 mm^3^, *P* = 0.033), while the tumor weight of SGC-7901/Lv-UBE2C group was heavier than that in SGC-7901/Lv-NC group (3.9 ± 0.62 vs. 2.7 ± 0.52 g, *P* = 0.026, data not shown).

### Up-regulation of UBE2C expression in gastric cancer was validated in a clinical cohort

**Figure 5:**
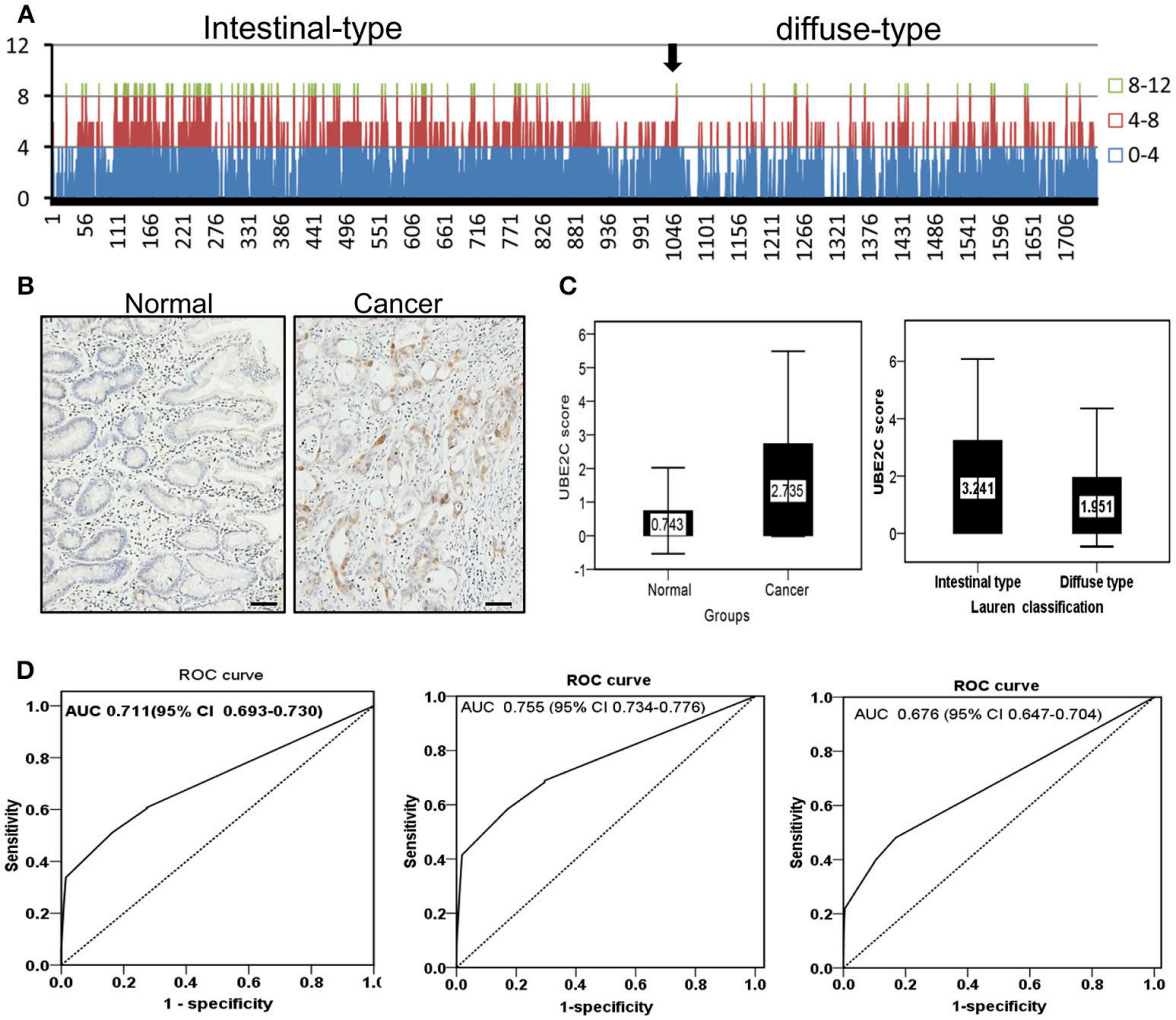
Protein expression and diagnostic value of UBE2C analyzed in a large cohort of gastric cancer. **(A)** Overall tendency of UBE2C expression in gastric cancer. Arrow indicates the borderline between intestinal-type and diffuse-type gastric cancer. The staining score ≥4 is dominantly observed in intestinal-type gastric cancer. **(B)** Immunohistochemistry. UBE2C protein is expressed in cytoplasm of intestinal-type gastric cancer, but negative in the corresponding normal mucosa. Scale bar indicates 25 μm. **(C)** The staining score of UBE2C is higher in gastric cancer tissues than in normal mucosa (left); the staining score of UBE2C is higher in intestinal-type gastric cancer than that in diffuse-type gastric cancer (right). **(D)** ROC curve of UBE2C in all gastric cancers (AUC = 0.711, left), in intestinal-type gastric cancer (AUC = 0.755, middle), and in diffuse-type gastric cancer (AUC = 0.676, right).

The protein expression of UBE2C was examined on tissue microarrays from a large cohort of gastric cancer by immunohistochemistry. The microarray set included 1,759 cases of cancer tissues and 1,710 cases were enrolled into final analysis because 49 cases was dropped during staining process. UBE2C was negative or weakly positive in normal gastric mucosa, while increased UBE2C expression was seen in gastric cancer, especially the intestinal-type gastric cancer (Figure 5A). The total staining score was higher in gastric cancer than that in normal mucosa (2.735 ± 2.709 vs. 0.743 ± 1.288, *P* = 0.001, Figure 5B) by semi-quantitative analysis. The staining score of intestinal-type gastric cancer was significantly higher than that of diffuse-type gastric cancer (3.241 ± 2.839 vs. 1.951 ± 2.409, *P* = 0.001, Figure 5C). The diagnostic value of UBE2C expression was evaluated by ROC curve analysis. The overall AUC was 0.711 (CI: 0.693–0.730, Figure 5D). The AUC of intestinal-type of gastric cancer was 0.755 (CI: 0.734–0.776, Figure 5D), and AUC for diffuse-type of gastric cancer was 0.676 (CI: 0.647–0.704, Figure 5D). It suggested that UBE2C could be a potential biomarker for intestinal-type gastric cancer.

**Figure 6:**
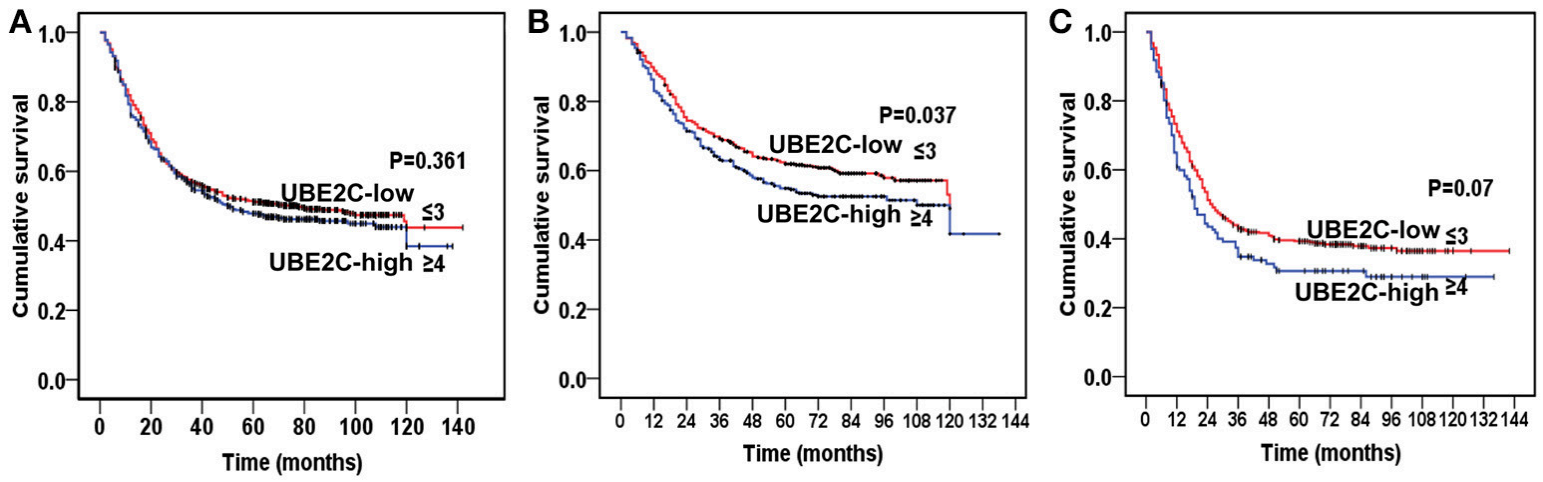
Kaplan–Meier analysis on prognostic significance of UBE2C expression. **(A)** All gastric cancers. There is no significant difference between UBE2C-high and UBE2C-low groups. **(B)** Intestinal-type gastric cancer by Lauren classification. The overall survival is significantly shorter in UBE2C-high expression group than that of UBE2C-low group. **(C)** Diffuse-type gastric cancer. The overall survival of UBE2C-high expression group is shorter than that in UBE2C-low expression group.

Among 1,710 cases of gastric cancer, 1,417 cases were used for survival analysis after a long-term follow-up. We divided cases into UBE2C-high group (staining score ≥4) and UBE2C-low group (staining score ≤3). There was no significant difference of overall survival between these two groups (*P* = 0.361, Figure 6A). However, when we separately analyzed intestinal-type and diffuse-type gastric cancer, the overall survival was significantly different between the two groups. In intestinal-type cancer, the UBE2C-high expression correlated with shorter survival than that in UBE2C-low expression group (*P* = 0.037, Figure 6B). In diffuse-type gastric cancer, the overall survival was slightly shortened in UBE2C-high group compared with the UBE2C-low expression group, but there was no statistical significance (*P* = 0.07, Figure 6C). These results indicated that UBE2C could be an oncogene related to intestinal-type gastric cancer.

## Discussion

Molecular classification of cancers has been achieved based on clusters of differential expressed genes using high through-put technologies. UBE2C is one of the genes used in molecular classification in several types of cancers. Studies revealed that overexpression of UBE2C is related to some kinds of solid tumors, such as stomach cancer (Zhang H. Q. et al., 2018), colorectal cancer (Cacciola et al., 2016; Zhang Y. et al., 2018), esophageal cancer (Matsumoto et al., 2014; Li et al., 2018), pancreatic cancer (Zhao et al., 2013), breast cancer (Mo et al., 2017; Qin et al., 2017), ovarian cancer (Martínez-Canales et al., 2018), hepatocellular cancer (Ieta et al., 2007), lung cancer (Tang et al., 2014; Zhang et al., 2015), prostate cancer (Shuliang et al., 2013), and other cancers (Wagner et al., 2004; Shen et al., 2013; Kraft et al., 2017). Therefore, UBE2C has been considered as an important functional gene in cancer development.

Lauren classification has been used over half a century. This classification was proposed by a Nordic pathologist based on microscopic observation (Lauren, 1965). Since there were no supporting technologies such as immunohistochemistry, molecular biology and gene sequencing, Lauren classification was a pure experience-based cancer classification. With the development of novel technologies, especially high-throughput methods, molecular classification has become possible. However, global genomic study is high-cost and there is always a limited case number in a single study. The experimental error was inevitable in studies from small sample size. In high-throughput community, submitting data was required for international file sharing. In this case, a large number of research data were uploaded in public database. Data mining of cancer-related data in public database could greatly reduce the cost and neutralize experimental error. Currently, huge amount of data were accumulated in GEO database of NCBI website and ArrayExpress database of EBI website (Barrett and Edgar, 2006; Parkinson et al., 2007). In this study, we focused on expressing profiles of microarray dataset of gastric cancer containing Lauren classification information, and found a set of Lauren classification-related genes. UBE2C is the gene we found that highly expressed in intestinal-type gastric cancer.

UBE2C gene is located on chromosome 20q13.12, which encodes one of the ubiquitin-binding enzyme families. Ubiquitin-binding enzymes are essential for activation of ubiquitin and binding to substrate proteins. UBE2C is a member of the anaphase promoting complex/cyclosome, and facilitates the degradation of target proteins with cell cycle progression (Nicolau-Neto et al., 2018). Therefore, UBE2C is an important regulatory factor of cell cycle. The imbalance of UBE2C expression could promote CIN and accumulated CNV of eukaryotic cells (van Ree et al., 2010; Heng et al., 2013; Sansregret et al., 2017).

In 2014, TCGA group reported a comprehensive molecular evaluation of 295 gastric cancers. They divided gastric cancer into four subtypes: tumors positive for Epstein-Barr virus, microsatellite unstable tumors, genomically stable tumors, and tumors with CIN. The latter shows marked aneuploidy and focal amplification of receptor tyrosine kinases (Cancer Genome Atlas Research Network, 2014). We recently reviewed histopathological images of those 295 cases of gastric cancer deposited in public database, and found that about 50% of gastric cancer in TCGA group belongs to CIN molecular subtype, which covered several phenotypes of intestinal-type gastric cancer with different degree of differentiation (Yu, 2018). In general, the overall survival of intestinal-type gastric cancer is better than that of diffuse-type gastric cancer (Liu et al., 2013). Molecular heterogeneity of cancer might explain the difference of biological behaviors of intestinal-type gastric cancer. Our study provided evidence that intestinal-type gastric cancer could be biologically heterogenous.

ERK1/2 signaling pathway is an important signaling pathway in tumor cells, which is closely related to immortality, growth factor-independent proliferation, apoptosis escape, cell cycle acceleration and cell invasiveness (Liu et al., 2010; Chen et al., 2013). It is generally believed that the degree of activation of ERK1/2 is positively correlated with the malignancy, and is related to the innate or acquired drug resistance to chemotherapy (Zhao et al., 2015). In our study, interfering UBE2C expression led to G2/M arrest. It was known that G2/M checkpoint is an important factor for cell cycle progression and drug resistance (Yang et al., 2016). Sarin and coworkers found that efficacy of cisplatin-based chemotherapy is limited by the occurrence of innate and acquired drug resistance. They examined lung cancer cell line A549 and its cisplatin-resistant sub-line A549rCDDP2000. Compared to A549 cells, the cisplatin-resistant cell line A549rCDDP2000 lacked cisplatin-induced G2/M cell cycle arrest, and led to reduced apoptosis (Sarin et al., 2017). Actually, many anti-tumor drugs act on cell cycle, most of them are strongly dependent on G2/M checkpoint, which suggests that G2/M checkpoint is a potential target for cancer therapy (Kawabe, 2004). Several years ago, Sabitha and Rajkuma have noticed the value of UBE2C as therapeutic target. They successful predicted two small molecular inhibitors *in silico* (Sabitha and Rajkumar, 2012). It is our hope to carry out some preclinical experiments for those small molecule inhibitors, which may provide a new strategy for targeted therapy of intestinal-type, a predominant subtype of gastric cancer.

In summary, this study identified a group of genes related to Lauren classification on the basis of data mining of public databases. UBE2C is one of the genes overexpressed in intestinal-type gastric cancer. We explored the biological function of UBE2C gene *in vitro*, and found that the overexpression of UBE2C is related to gain of DNA copy number caused by CIN of intestinal-type carcinoma. Overexpression of UBE2C accelerates cell cycle progression and promotes cell growth and invasiveness of gastric cancer cells by activating ERK1/2 signaling pathway. The increased expression of UBE2C of intestinal-type gastric cancer is verified in a large cohort of gastric cancer tissues from Shanghai, China. It was found that overexpression of UBE2C could be the underlying molecular mechanism of biological heterogeneity of intestinal-type gastric cancer and overexpression of UBE2C correlated with poor overall survival of intestinal-type cancer.

## Author contributions

JZ, XL, GY, ZZ, and YY conceived the original idea and designed the study. GY, JW, XC, YB, YJ, XZ, JF, YS, and HC provided samples from multiple medical centers. LL, YC, JJ, ZX, and LG performed experiments. JZ and YY performed data analysis and manuscript writing. All authors contributed to data interpretation.

### Conflict of interest statement

The authors declare that the research was conducted in the absence of any commercial or financial relationships that could be construed as a potential conflict of interest.
